# The Closing Scene
*The Closing Scene; or, Christianity and Infidelity contrasted in the Last Hours of Remarkable Persons. By the Rev. E. Neale, M.A. London, 1849.


**Published:** 1851-04-01

**Authors:** 


### THE JOURNAL

OF

### PSYCHOLOGICAL MEDICINE

AND

### MENTAL PATHOLOGY.

APRIL 1, 1851.

### Art. I.—THE CLOSING SCENE.*

We consider the subject before us to involve points of deep and solemn interest. Having special reference to the mysterious union of mind and body, spirit and matter, we do not think it to be foreign to the original scope and design of this journal.

The pathologist and physician cannot, as they are constantly called upon to do in the exercise of their professional duties, witness the "closing scene" without serious reflection on the mutability of human life, and the awful beauty and sublimity of a Christian's death-bed, and the hopeless misery, alas! so often associated with the last struggles of those who have repudiated the great truths of Christianity. It is at this awful moment, when, according to the creed of the believer, the spirit is on the point of winging its flight from the body, that the nakedness of the heart is displayed. It is then that the solemn vista of futurity opens to the unclouded vision, and the immortal spirit, concentrated in its self-judgment, acknowledges all its depravity, for once, perchance, too late. Alas! that in the heyday of his pride and ambition something like this "clairvoyance" came not over man's heart, to be a lamp to his path; that he had not "remembered his Creator in the days of his youth, when the evil days came not;" *now* he has no pleasure in them. "*Nihil est in morte,*" writes one of the Fathers; "*quod metuamus si nihil timendum vita commisit.*" This thought must constantly be passing through the mind of the physician in the performance of his solemn duties at the bed-side of

* The Closing Scene; or, Christianity and Infidelity contrasted in the Last Hours of Remarkable Persons. By the Rev. E. Neale, M.A. London, 1849.

his dying patient. The awful contrast between *now* and *then*—the studied neglect of the past—the dread penalties even at the present, and—what of the future! must be the frequent subject of his meditation.

But a still more solemn office is often imposed upon him. He becomes, of necessity, the confessor of the penitent; the secret errors of many a past life are unfolded to him; for the dying, reluctant to summon formally the minister of religion to his bed, to listen to his confession, displays to his confidential physician, even if there be no direct confession, yet in the statement of his symptoms and sensations, the courses of his crimes. These vital errors are often, indeed, the sources, the exciting causes, of his malady.

It is by his reflection on these recitals that the physician is led to contemplate the causes, not only mental, but corporeal, of these delinquencies; and without for a moment doubting the sinfulness of man's original nature and the wiles of an evil spirit, the truth of which sacred writ has revealed to us, he becomes more and more sensible of the influence of morbid action, if not in *originating* an evil thought or deed, at least in reducing the organic frame to a condition in which it more readily becomes the prey of temptation. As in the animal and vegetable kingdom the low degree of vitality is constantly intruded on by the development of the parasitic germ, so the disordered frame is too often rendered morbidly impressible, incapable of resisting vicious impulses, which the flush of pure health might often avert, even by its real sense of the more healthful pleasures of existence.

The happy, because innocent, heart is known by its laughing eye and dimpled cheek. How important is it, then, to look to these days of youth, when even to live and breathe is happiness, at the moment when, by preserving health, we may form one bar at least to those slavish passions that will in the end cast a cloud of gloom over the closing scene.

The question of the *responsibility* of the human being is too sacred to discuss in this journal, but, with our deep acknowledgment of the solemn truth, we as confidently feel that crime *may be* averted by the judicious treatment of the body, not only regarding the cerebral but the abdominal, and even the thoracic organization. Thus are we naturally led to study those conditions which may be said in some degree to involve the etiology and pathology of actions termed sinful. When we presume to term sin, the commission of crime, often an act of a diseased mind, we do not diminish one atom the responsibility of our being, indeed the insanity itself is too often a crime, *because it is fostered, if not induced, by a man's own sinful indulgence, a self-created condition* for which he will be made accountable.

If, in our allusion to the prophylaxis of error and of crime, we turn our thoughts naturally to childhood, we do not, for a moment, lay a paramount stress on the value of the materia medica in preventing the development of the germ of evil, or in stamping that character which may confer a blessing or a bane on the closing scene of life. We allude to the necessity of the health of the soil. It is, of course, *moral* culture which is then eminently influential in forming character. It is that which keeps the brain healthy, controls bad impulses, regulates and directs thought and action in the right path. Nay, we concede thus much, that, even with some defect of organization, moral culture may constantly control its evil workings. Else, if organization, the theme of speculative phrenology, were all in all, education would be in a great degree valueless; weeds would be allowed to grow and choke the flowers of intellect—the mind would be worse than a wilderness.

And how deeply, then, is the mother concerned in this! By her wisdom in the nursery many a noble creature has been modelled who has dignified our nature, and blessed society. By the unwise parent, who has presented a bad example, or who has committed a scarcely less venial fault, the direful error of correcting the child according to *feeling* and not *judgment*, many a being has been doomed to a life of sin and sorrow, and to a closing scene marked by agony and despair; lamenting, perhaps cursing to the last, either the false indulgence or the cruelty of his nursery life. History and our own experience are prolific in examples of the good and bad effects of such early influences.

Jane Taylor's mother, we are informed by the author, taught her early to read aloud good books; and the father of John Foster was a man of energetic mind, and constantly assembled in his house his Christian neighbours, for the celebration of social devotion. The mighty mind of the great Alfred, we are told, was excited by his mother's gift, and we may believe the share she might claim in its subsequent purity and dignity.

Depraved examples and bad precepts exist by so much more influence, as the heart of man naturally leans towards evil, and, moreover, sin gratifies *at once* the passions and appetites, while virtue, although acknowledged to be the very handmaid of happiness, must constantly practise self-denial, and merely point in perspective to the future. The wayward existence of Byron, as we gather from Moore, was sadly influenced by the erroneous management of his mother in his youth. Lord Ferrars, whose impetuous passion at length incited him to murder, was petted and left to his own guidance; but his mind was naturally wayward, and it was self-sacrificed. Not the slightest mark of his depravity was *his own* cross examination of witnesses to prove himself insane! and yet, one of his last sentences was, "In doubt I live, in doubt I die!" We can scarcely look on a more melancholy picture. Lord Camelford, the great *shot*, was in his youth permitted, almost encouraged, to seek cause for quarrel and the duel. His mind was deeply tainted by sceptical books. Thus shall we find mature life and the closing scene foreshadowed as it were by the unrestrained derelictions of childhood. Then the course of study—even Beckford's polished mind, 'tis said, was perverted by the purchase of Gibbon's library, and the ardent contemplation of his marginal notes. Still, though there were inconsistencies in Beckford's life, we cannot place him in the category of debased scepticism. The refined mind that yet never seemed to suffer a moment of ennui, that directed the daily offering up of prayers, or the celebration of mass, could scarcely be depraved: and although his closing scene was silent and placid, only without the *expression* of devout faith in the merits of redemption, his self-penned epitaph proved him to have really died in hope.

The casualties of life also, as short-sighted mortals term them, how influential are they in modelling, sometimes in metamorphosing, the characteristics of a life: the loss of valued friends, for instance. The religious impressions of Madame de Stael, after her family misfortunes, became more deepened, and her sleepless nights were spent in prayer. She became convinced of that truth which is one of the chief inducements to virtue and humanity, even in the worldly-minded, the *natural* consequence of punishment to crime. "I never," she writes, "committed an error that was not the cause of disaster." Thus what earthly thought may deem an infliction, the Christian will feel to be a blessing: for however severe may seem to be the visitations of the Deity at the moment of our suffering, we may be assured, that when the scene of life is closing, we shall, if we have read the divine lesson aright, reflect on them as among the blessings of our existence. Even the idle prophecy, as it seems to us, may not be without its permanent influence. The young heart of Elizabeth Fry was the seat of extreme personal vanity; but when she had listened to Deborah Darby's prognostication of her philanthropy, she was at once converted from the ornamental to the useful, and became, in the words of our author, "the helper of the fallen."

Even one sentence may be an all-ruling impetus. The munificence and posthumous charities of Edward Colston, of Bristol, are said to have been incited by the axiom of the papists of Spain, that the life of no *reformed* religionist was ever consecrated by philanthropy. Thence did Colston shine forth as the honoured companion of William Canynge and Thomas Guy, and other merchant princes of our land, with Mrs. Partis, "the munificent churchwoman," as our author terms her.

The contemplation of the varied modes of meeting death forms a subject of curiosity even to the most thoughtless and ignorant: it is a constant theme among the crones of the village, and, indeed, the most depraved members of society. The "wisest of mankind" has thus written. Augustus Cæsar died in a compliment—"*Livia conjugii nostri memor vive et vale;*" Tiberius, in dissimulation, as Tacitus saith of him—"*Jam Tiberium vires et corpus non dissimulatio deserebant;*" Vespasian, in a jest—"*Ut puteo Deus firo;*" Galba, with a sentence—"*Feri si ex re sit populi* Romani;" holding forth his neck, Septimius Severus in despatch,—"Adeste *si quid mihi restat agendum;*" and the like.

But to the philanthropist the contrast of the closing scenes of the devout and the infidel affords a theme beyond that of mere curiosity, nay, of sublimity. We do not think, however, that Neale has been happy in the arrangement of these comparisons, and we might have hoped for something like a psychological analysis of the quiet death-bed of the mere moralist. The comparison between the *great* Frederick, who, as Zimmerman writes, "died in a continued disbelief of revelation, and even of the immortality of the soul," and our fourth William is a fallacy. We might easily point to examples which would better have illustrated the subject, and displayed the deformity of Vice, the beauty of Virtue, in much higher relief.

The generosity of a sceptic, who seems to love his neighbour as himself, merely from his desire to relieve, must of course be appreciated by all; but the quiet repose of the mere utilitarian should have been shown to differ as much from the happy falling to sleep of the righteous, who "makes signal of his hope," and whose every thought and word is consecrated by the odour of sanctity, as the cold and hopeless sleep of an eternal grave is eclipsed by the blissful life to come of the angelic spirit. Else must Christianity be shorn of that beautiful halo of purity with which faith encircles the life and death of the righteous.

We are told that Talleyrand's end was peaceful and quiet; that Mirabeau desired music to send him to the sleep from which there was "no awaking;" that Bentham, whose life was passed in writing to ensure "the greatest happiness of the greatest number," died quietly, almost imperceptibly, on Dr. Bowring's bosom; that Theodore Hook, who was constantly in debt and difficulty, his diplomatic character in the shade, left the world peaceably, as did also Bolingbroke, who asserted that "there was no such place as heaven," with his last breath; that Tom Paine went off calmly, with a joke about Charon on his lips, and this confession to the reprobates who came to see him, "I have no wish to believe in Christ."

Now, we are all aware that any potent or all-absorbing passion may seem even to overcome the fear of death: but we believe that we are not here in possession of the whole truth. We know, for instance, that Paine, with all his vaunted calmness, courage, and contempt of death, was often wont to give most audible expressions to his terror in solitude, under the impression that he saw spectres; and, when indisposed, cried out, "Oh, Lord, help me! Jesus Christ, help me!" We are aware, too, that, from other free-thinkers, extreme peril will bring out the truth in spite of resolution. Volney, when in great danger of shipwreck on a North American lake, exclaimed (as we learn from Mr. Bancroft), "Oh, mon Dieu!" but when the peril was over, as if ashamed of his confession, he recanted, and said with the fool, "There is no God."

Now, we believe we might easily prove, too, that, in a seeming *placid* dissolution, there is often a sort of slavish and Satanic pride—a determination to *die game:* such was the impersonation of evil in Maria Manning, somewhat analogous to the bravado of the convulsive laughter of Mandrin on the wheel, which, to the outward gaze, cloak the real emotions of the dying heart. Thistlewood, on the night preceding his execution, while he thought his keeper was asleep, fell on his knees, and prayed to God through Christ; but the proud and obdurate heart afterwards denied him on the scaffold.

But this prospectless sinking into death, and, as the sceptic believes, into absolute annihilation,—can this be compared to the sleeping of the faithful—the passing from a life of sorrow to one of endless bliss, full of that hope which is the very rainbow of the soul? The truth is at once confessed, and we need do no more than point to the contrasted sketches of the author, slight as they are, as illustrations.

Look to the closing scene of two children of genius, for instance—Theodore Hook and Felicia Hemans, (her name so aptly foreshadowing the passage of her pure spirit from this world.) The highest eulogy ever passed on the most sparkling sallies of the wit must have yielded a thousand-fold to the intensity of delight, when the *stranger* told *her*, almost on her death-bed, that she had *converted him from atheism* by her poem of the "Sceptic."

We may adduce, too, the contrast between Paine and Locke; the vain and boisterous implorings of the one, and the beautiful confidence in a happy future displayed by the other. We may point to the closing scenes of Addison and of Walter Scott, whose lives were full of worth—that of Scott, especially, being one happy course of joyous gratitude to his Creator, and of love to his neighbour. We remember the brief but impressive precepts imparted by each on his death-bed to his son-in-law. When Addison showed the young Earl of Warwick "how a Christian can die;" and Scott whispered to Lockhart, "My dear, be a good man—be virtuous—be religious—be a good man. Nothing else will give you any comfort when you come to lie here."

On these gifted mortals, on the last especially, were bestowed the eulogies of half the world, and yet the glory of their earthly fame did not on their dying bed even enter their thoughts; these, full of hope and joy, were fixed on eternity.

The physician, we may without presumption believe, with his experienced study of physiognomy and expression, *must be* more deeply read than the divine in the *interpretation* of the closing scene, in the appreciation of these interesting contrasts.

Beyond, far beyond the voluntary expression of the lips, which, as in the instances to which we have alluded, may be, according to the wily Frenchman, uttered to conceal our thoughts, is the faculty of the *endurance* of pain. This has been wonderfully exemplified in the Christian, whose hope of a better state confers a giant strength of endurance beyond all which the anæsthetic influence of chloroform can impart. He deems this a probation, as we have hinted at before, rather than an infliction; or, if human nature for a moment subdues his spirit, he will soon rally, and exclaim with his Redeemer, "Thy will, not mine, O Lord, be done."

And in the endurance of life, too (for the fear of living is often as great as the fear of dying), how does the Christian soar above the evils of the world? He is the hero: the suicidal stoic is the coward. The imperial Julian said as he was dying, "He that would not die when he must, and he that would die when he must not, are both of them cowards alike." It is this triumph of soul that the constant and repeated visitation of the physician, combined with his fortitude and his *concentration* of mind, can so fully appreciate. He has listened to the agonizing groan of the dying unbeliever, and to the holy aspirations of the devout; he has gazed on the remorseful contortions of sin (for even the *laugh* of the hardened spirit more resembles the glare of a demon than of a human expression), and on the seraphic smiles of dying innocence; and, with all this evidence, he must have felt that a life of denial, and even of suffering, is beyond all measure repaid by the Euthanasia of a believer. Few fear the transit of death but those who deem this world happier than the next.

The physician thus watches, attentively, the hour of birth and of the closing scene, and he hence unconsciously becomes a psychologist. He sees how intimately blended are the development of mind and body, how deep the effect of mental influence on the frame, of the controlling effect of health and disease on the intellect and passions, and he is thus led to institute analysis, and form deductions, often beyond the scope of the divine and the moralist, who too often argue hypothetically, because without the light of *physical* psychology.

The author of this book, with all his charity, has unhappily become tainted with the unjust fallacy of some baneful influence, essentially imparted by *physiological* study: and, in answer to the remonstrance of Dr. Hall, of East Retford, against the stigma of medical infidelity, aims at the justification of his libel by quoting the crude regrets of a young medical student to Mrs. Fry. We might have thought that the author's own selection of James Hope, whose whole life was devoted to the most *material* prosecution of morbid anatomy, as the converse of the infidel Volney, would have led him to a fairer conclusion. We might at once prove the deep injustice of this anathema, by pointing to the daily exercise of *practical* Christianity by the profession. And what, we would ask, tends more to raise our wonder and admiration of the Creator, than the contemplation of that structure which was fashioned in his own image? Thus to "look through Nature up to Nature's God," is a practical lesson of devotion. Yes,

"An undevout *anatomist* is mad :"

He cannot for a moment reflect, without the conviction that an Almighty power, that instituted the laws of nature, is still overlooking their wondrous operation, and thus the holy groundwork of *natural* religion is at once instilled into his very being. Let us look to the fruits, and, without invidious feeling, point to the hospital, the university, and the inns of court—their studies. In the one, the elucidation of Nature's truth is sought, not for the mere idle pleasure of contemplation or barren discovery, but that the consequent deductions may become blessings to mankind. In another, the mind is, of necessity, deeply imbued with the licentiousness of the heathen writers, Ovid Virgil, Horace, Anacreon, Catullus. And can it be that, with all the counterbalance of the *moral* writers of antiquity, with all the heroic virtues of the Roman stoic, whose creed was fashioned before the Advent, can it be, that the Divinity lecture, in a young and glowing heart, will often countervail the poison which the loose pictures that the Classics, the paramount study, hold up to the passions?

But we may advance a still higher claim. Religion is now one of the handmaids of our hospitals; and we may indeed hope, that devotion will, ere long, form one prominent feature of the curriculum of our academical study. The religion of medicine may then shine with almost as bright a light as the religion of tracts and homilies.

We believe that our author, and many a Christian advocate before him, have formed their erroneous notions from the perusal of those physiological disquisitions which refer the *evidence* of mind or soul to organization. We might point here to many a learned combat regarding the nature and relations of spirit and matter, but we waive at once our refutation of this one-sided criticism.

On this point, however, our author becomes bewildered on the very threshold of his metaphysical psychology. Materialism—the word materialism is the scarecrow of his mind, and, in his dread of its contamination, he will have it that body—that is, organization—is to serve merely *earthly* purposes, and has nought to do as a medium of the spirit's communication.

In his alarm, too, he forgets, or he has never learned, that a simple idea, by exaggeration, becomes a phantom: a thought and a ghost differ only in *degree.* He is referring, for instance, to a mere vision of Shelley's excited intellect, and confesses, "there are incidents in his life for which we seek vainly an explanation."

But will the medical psychologist join in this confession? If he will, and thus flies from the patient investigation of secondary causes, he neglects his high and almost sacred duty, and at once forfeits his title, dwindling down from the scientific physician to the shallow empiric or the self-blinded bigot. He may, perchance, point in triumph to the miracles of Hohenlöhe, and say, "Behold the wondrous power of faith!" The physician meets him on his own ground, and at once coincides as to the *fact.* But *how* has faith *worked* the miracle? Is it not on the principle of imparted confidence, of which happy influence on disorder our note-books teem with illustrations?

But the study of physical psychology looks deeper still than this. It is not for the mere incitement to triumphant discovery, but with the Christian hope of inducing that health of mind, that may insure a happy life on earth, and, what is of infinitely more importance, Euthanasia of the closing scene.

We believe, wherever there is a phantom—a waking or a dreaming ghost—there is a certain change or action of the brain. This may be *healthy* action, but it is often, most generally, morbid: it is often removed, like the delirium of fever, by mere depletion; the ghost passes out with the blood, and vanishes from the sight as the consequence of a healthy cerebral circulation. Nay, the gush of blood from the jugular or carotid has, in a moment, even cured the insanity of the suicide, when, alas! it was too late to awake to reason! This has been proved in more than one prominent instance, and reads an awful lesson to our watchfulness and judgment in these cases. Let Mr. Neale, then, undeceive himself that the Psychologist, like the Divine, seeks *vainly* for the *explanation,* or even the *laying* of the ghost, and let him allow that it is as much *our* duty to study and elucidate psycho-pathological causes, to minister to the health of the mind, *by the medium of its*

*organ,* as it is that of the Divine to minister, by precept and gospel elucidation, to the welfare of the unfettered spirit.

It is through or by the medium of organization that the immortal essence, when on earth, must display its social intercourse. It is in and by the body that the soul commits those crimes which cast a gloomy shadow over the closing scene, and doom it to everlasting pains.

How all-important, then, the study of pathology, even in regard to our moral being: how essential, above all, to minister to the health of that brain which, when disordered, we believe to be the source and seat of those maladies which often lead to frenzy and to lunacy, and the immediate spring of those wayward passions which, when uncontrolled, entail sorrow and suffering on many an unhappy being throughout a restless life—and—eternity.

It is not essential, however, in our desire to bring pathological science to the aid of morality and religion, that we should confine our study to the brain: passions may spring not only from idiopathic cerebral affections, but also from remote irritations, that act through the medium of the brain by its intricate sympathies.

Valvular and hypertrophied disease of the heart may at once completely change the disposition. Of this we have an instance, even while we are writing: the mind of a most amiable and patient gentleman changed into a hasty and querulous spirit. His heart is concentrically hypertrophied, and the mitral valve deranged. When protracted, disease of the generative organs may incite to inordinate and uncontrollable passion; and hepatic or gastric disorder may induce melancholy, with all its train of moral and physical evils. We may safely affirm, then, that it will very often be vain for the moralist to presume on his lessons and his prayers for the conversion of an erring mortal, while a material excitement is goading on the deranged body to a crime.

How often, moreover, is painful disorder the inducement to the abuse of alcohol or opium, at first an innocent anæsthetic, but which, step by step, growing by what it feeds on, terminates in confirmed and slavish drunkenness. The moralist doles out his dissuasive precepts but to be ridiculed or despised, while the poison is again and again sought: and no wonder, as it again laps the suffering mortal for a time in Elysium. The seed, in itself inestimable, falls on worse than barren ground, for it is choked by the weeds of disease.

Yes, the moralist and the divine *must* often wait for the skilful husbandry of the psychological physician to root out the malady that poisons or weighs down the spirit, to restore the mental soil to a healthy condition, by which the good seed of their discourse may germinate and grow to a prosperous maturity.

With all this, we must yet acknowledge the power of a holy life in controlling disease and pain. It has been maintained, and we think with truth, that under the infliction of acute disease the Christian has, *cæteris paribus*, the best chance of recovery. His belief in the consoling and calming doctrine of the Bible, tranquillizes the mind, and thus gives power to the operation of the *vis medicatrix Naturæ*. Kirke White bore his sufferings with exemplary patience, and Wollaston studied pathologically his own closing scene.

But, in conclusion, we may remark, that even here the truth of cause and effect is not always developed, except to the physiologist. In phthisis, the words and sentiments of the young consumptive are often all but angelic. How far a material cause, the high oxygenation of the blood, is influential in inducing this peculiar temperament, we must not now inquire, but if we watch to the close the subjects of phthisis and dyspepsia, we shall be instantly sensible of the fact to which we have glanced.

We trust that we have offered by our brief remarks a sufficient apology for having converted so sacred a subject to our present purpose, and have proved that the duty of the physician is of the deepest importance, even in the prophylaxis of crime, and in its influence upon the closing scene.

---

